# Boron-doped Nanodiamond as an Electrode Material for Aqueous Electric Double-layer Capacitors

**DOI:** 10.1038/s41598-019-54197-9

**Published:** 2019-11-28

**Authors:** Kenjo Miyashita, Takeshi Kondo, Seiya Sugai, Takahiro Tei, Masahiro Nishikawa, Toshifumi Tojo, Makoto Yuasa

**Affiliations:** 1 Department of Pure and Applied Chemistry, Faculty of Science and Technology, Tokyo University of Science, 2641 Noda, Chiba, 278-8510 Japan; 2 Daicel Corporation, 1239 Shinzaike, Aboshi-ku, Himeji, Hyogo 671-1283 Japan

**Keywords:** Electrochemistry, Batteries

## Abstract

Herein, a conductive boron-doped nanodiamond (BDND) particle is prepared as an electrode material for an aqueous electric double-layer capacitor with high power and energy densities. The BDND is obtained by depositing a boron-doped diamond (BDD) on a nanodiamond particle substrate with a primary particle size of 4.7 nm via microwave plasma-assisted chemical vapor deposition, followed by heat treatment in air. The BDND comprises BDD and sp^2^ carbon components, and exhibits a conductivity above 10^−2^ S cm^−1^ and a specific surface area of 650 m^2^ g^−1^. Cyclic voltammetry measurements recorded in 1 M H_2_SO_4_ at a BDND electrode in a two-electrode system shows a capacitance of 15.1 F g^−1^ and a wide potential window (cell voltage) of 1.8 V, which is much larger than that obtained at an activated carbon electrode, i.e., 0.8 V. Furthermore, the cell voltage of the BDND electrode reaches 2.8 V when using saturated NaClO_4_ as electrolyte. The energy and power densities per unit weight of the BDND for charging–discharging in 1 M H_2_SO_4_ at the BDND electrode cell are 10 Wh kg^−1^ and 10^4^ W kg^−1^, respectively, and the energy and power densities per unit volume of the BDND layer are 3–4 mWh cm^−3^ and 10 W cm^−3^, respectively. Therefore, the BDND is a promising candidate for the development of a compact aqueous EDLC device with high energy and power densities.

## Introduction

Supercapacitors or electrochemical double-layer capacitors (EDLCs) are electrochemical energy storage devices. Charging–discharging of EDLCs is based on adsorption–desorption of electrolyte ions and not on faradaic current, thereby enabling faster charging–discharging performance and longer lifetimes for EDLCs than for secondary batteries^1–4^. However, EDLCs possess low energy density; therefore, enhancing their energy density is vital for practical applications. The energy density of an EDLC can be calculated as *E* = *CV*^2^*/2*, where *E*, *C*, and *V* are the energy density, capacitance, and cell voltage, respectively, which indicates that both the capacitance and cell voltage should be increased to obtain higher energy density. In this context, organic electrolytes are typically used to generate a large working cell voltage; however, organic solvents have some limitations such as high cost and low conductivity^3^. Since the use of aqueous electrolytes is considered to overcome these limitations, the development of aqueous supercapacitors with large cell voltage is highly desirable^4,5^.

Boron-doped diamond (BDD) is a conductive material and a candidate electrode material for EDLCs with large cell voltages because it exhibits a wide potential window even in aqueous electrolytes^6–8^. To use BDD as an electrode material for EDLCs, its specific surface area should be increased to improve its capacitance. Several studies have reported BDD materials with improved specific surface areas^9–21^. In our previous study, we used BDD powder (BDDP) as electrode material in an aqueous EDLC, which was prepared by depositing a BDD layer on the surface of an (insulating) diamond powder substrate. A larger specific surface area was expected as the powder diameter decreased, leading to an EDLC with larger capacitance. We reported the use of BDDP as an electrode material for printed electrochemical sensors^22–25^ and polymer electrolyte fuel cell cathode catalyst support^26^. In these reports, the diameter of the BDDP was 300 nm or larger. It was also applied to prepare an aqueous EDLC, and the effect of its size (150, 350, and 3500 nm) on the electrochemical properties of the BDDP was investigated. The capacitance of the BDDP was found to increase upon decreasing the diameter. Furthermore, cyclic voltammetry (CV) measurements performed with a symmetric two-electrode cell showed a large cell voltage of 1.5 V in 1 M H_2_SO_4_, whereas that for activated carbon (AC) was 0.8 V^27^.

Herein, we describe the fabrication of a BDDP, namely boron-doped nanodiamond (BDND), with a diameter of approximately 100 nm or smaller to improve its specific surface area. In another study, BDDP with a diameter of 100 nm or smaller was fabricated by crushing the BDD film^28^. Instead, we fabricated the BDND by depositing a BDD layer on a detonation nanodiamond (ND) with a primary particle size of 5 nm as a substrate material using microwave plasma-assisted chemical vapor deposition (MPCVD). The electrochemical properties of the BDND were investigated toward its application as an electrode material for an aqueous EDLC with large cell voltage.

## Results and Discussion

### Preparation of the boron-doped nanodiamond

**Figure 1:**
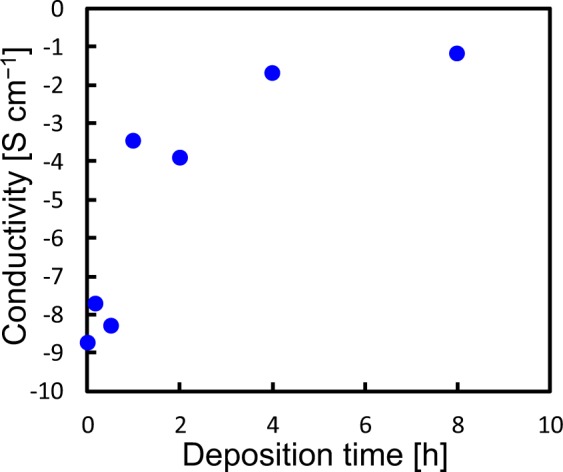
Electrical conductivity of the as-deposited BDND as a function of the CVD deposition time for the BDND preparation.

To determine the deposition time of the MPCVD process for the BDND preparation, the electrical conductivity of the BDND before heat treatment was measured as a function of the deposition time (Fig. 1). The electrical conductivity of the as-deposited BDND was increased rapidly from 1.8 × 10^−9^ S cm^−1^ to 3.5 × 10^−4^ S cm^−1^ with a deposition time of 1 h. The BDND prepared with 8 h deposition time exhibited a conductivity sufficient for an electrochemical electrode material (2.0 × 10^−2^ S cm^−1^). Therefore, we set the MPCVD deposition time for the preparation of the BDND as 8 h.

**Figure 2:**
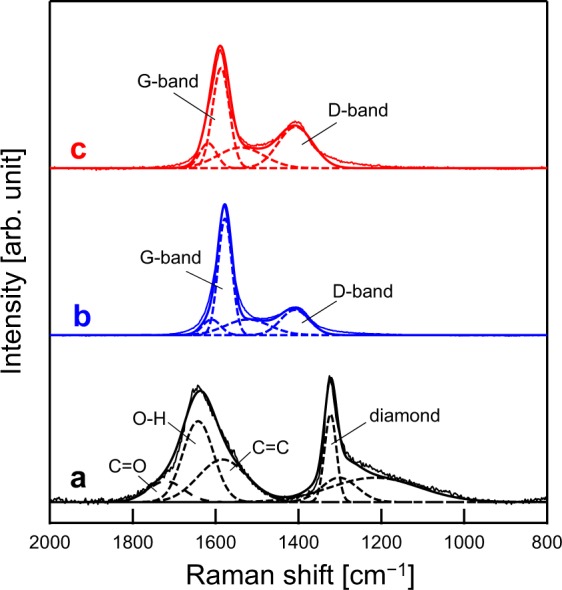
UV Raman spectra of the (**a**) ND, (**b**) as-deposited BDND, and (**c**) heat-treated BDND. Solid thin and thick lines indicate experimental and simulated curves, respectively. The spectra were decomposed into Voigt profiles (dashed lines).

The UV Raman spectrum of the ND showed a sharp peak at 1325 cm^−1^ and a broad peak at around 1650 cm^−1^, as similarly reported by Mochalin^29^ (Fig. 2a). The peak at 1325 cm^−1^ can be deconvoluted into a main peak at 1320 cm^−1^ and a shoulder peak at 1220 cm^−1^ with a small unknown peak at 1300 cm^−1^. The peak at 1625 cm^−1^ was found to be a combination of three bands at 1580, 1640, and 1720 cm^−1^, which were attributed to graphitic carbon, OH bending, and C=O stretching originated from surface functionalities^29^. In the case of the as-deposited and heat-treated BDND, broad peaks at approximately 1410 and 1580 cm^−1^ for D and G bands, respectively^30^, were observed, which indicated that a significant amount of sp^2^ carbon components existed even after the heat treatment (Fig. 2b,c). Since the presence of the sp^2^ carbon structure may be detrimental for the electrochemical properties of the BDD (e.g., wide potential window), heat treatment in air at 425 °C was performed to remove the sp^2^ carbon impurities.

**Figure 3:**
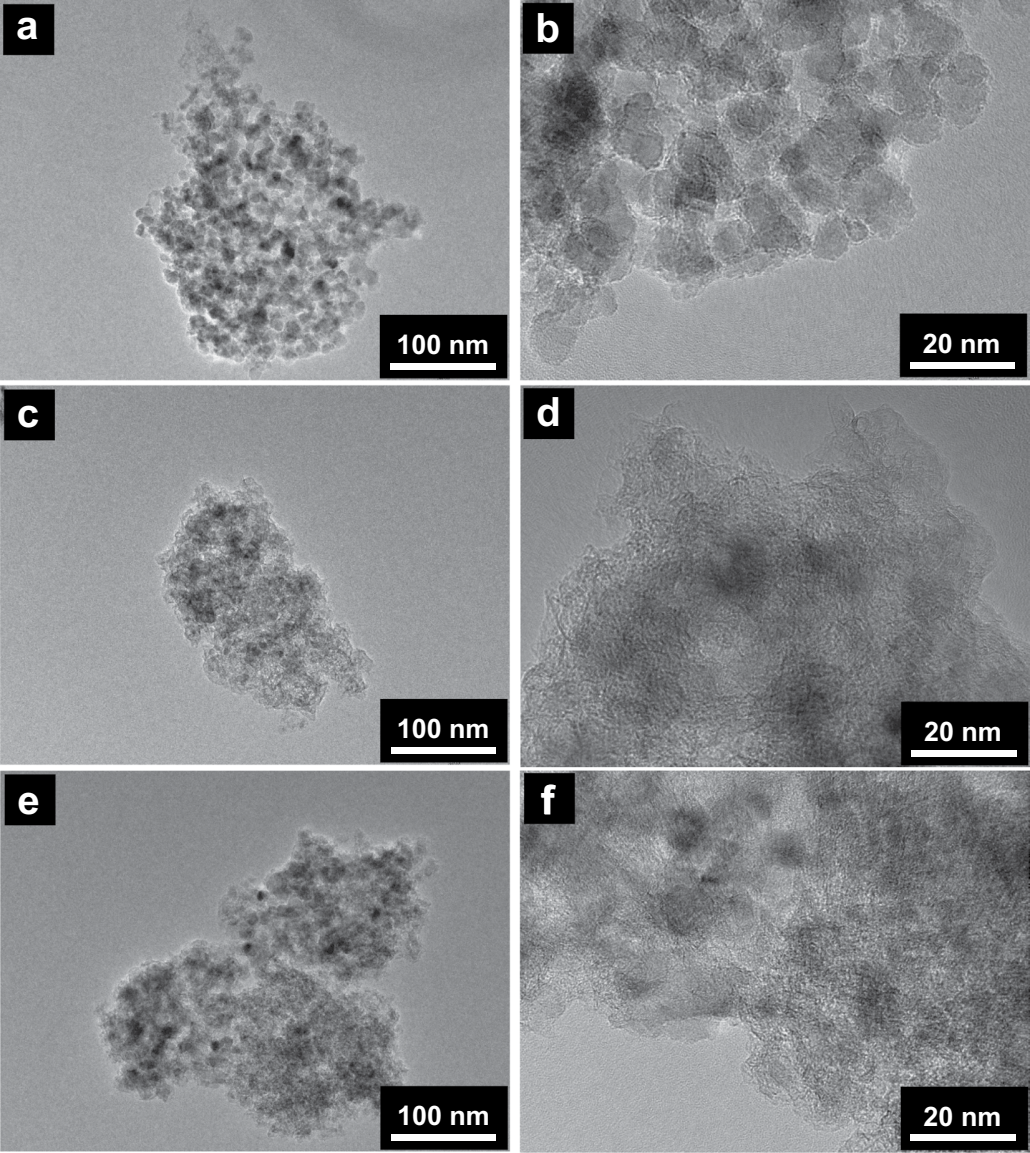
TEM images of the (**a**,**b**) ND, (**c**,**d**) as-deposited BDND, and (**e**,**f**) heat-treated BDND.

Figure 3 shows the transmission electron microscopy (TEM) images of the ND, as-deposited BDND, and heat-treated BDND. The diameter of the ND was 5–10 nm, and the lattice spacing was determined from the image to be 0.2 nm, which is in agreement with the diamond (111) planes^31^ (Figs. 3a,b, S1a and S2a). In the image of the as-deposited BDND, although primary particles could not be clearly seen, the lattice spacing of 0.2 nm for diamond and a layer spacing of 0.367 nm attributable to graphite were observed (Figs. 3c,d, S1b and S2b). This layer spacing was slightly larger than that reported for graphite (0.335 nm)^32^, which can be attributed to the lower crystallinity of the graphitic components. From this result, the CVD process can be considered to give rise to graphitic components covering the particle and deposition of the BDD on the ND agglomerate. After heat treatment in air at 425 °C for 8 h, the graphitic component was partly removed, resulting in the exposure of the diamond surface (Figs. 3e,f, and S1c). The average diameters obtained by dynamic light scattering measurement of the as-deposited and heat-treated BDND were 111 and 120 nm, respectively. Elemental analysis using inductively coupled plasma atomic emission spectrometry (ICP-AES) revealed that the boron concentration increased from 440 to 620 ppm after the heat treatment. Since the BDND has an (undoped) ND core, the boron concentration in the BDD layer is considered to be larger than these values.

**Figure 4:**
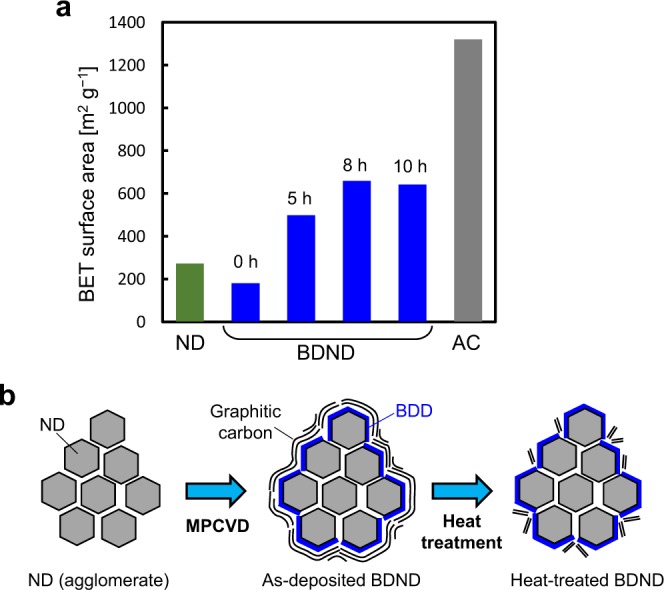
(**a**) BET specific surface area of AC, ND, and BDND with various heat treatment times (0, 5, 8, and 10 h); (**b**) A schematic illustration of the BDND preparation.

Figure 4a shows the Brunauer–Emmett–Teller (BET) specific surface area estimated from the nitrogen gas sorption measurement of the BDND sample after heat treatment in air for various time periods following the CVD process. The as-deposited BDND exhibited a BET specific surface area of 182 m^2^ g^−1^, which increased remarkably to 658 m^2^ g^−1^ after heat treatment in air at 425 °C for 8 h. The specific surface area was almost the same after 10 h of heat treatment; therefore, heat treatment for 8 h was found to be the optimum condition. This increase in the specific surface area after heat treatment is most likely due to removal of the sp^2^ carbon components contained in the BDND. The as-deposited BDND consists of an ND agglomerate covered with BDD and sp^2^ carbon materials, which is expected to cause a decrease in the specific surface area from that of the original ND. However, the heat treatment would remove the vulnerable sp^2^ carbon part, leaving a stable sp^2^ carbon structure, e.g., a graphene-based structure. This is supported by the fact that the Raman spectra of the BDND before and after heat treatment were similar (Fig. 2b,c). The remaining sp^2^ carbon structure should contribute to increase significantly the specific surface area (Fig. 4b). Although the sp^2^ carbon components could not be removed completely from the as-deposited BDND with heat treatment for 8 h, the remaining sp^2^ carbon components can be considered to contribute to the large specific surface area, which was almost half of that obtained for an AC sample used in this work, i.e., 1318 m^2^ g^−1^.

### Electrochemical investigation for aqueous supercapacitors

**Figure 5:**
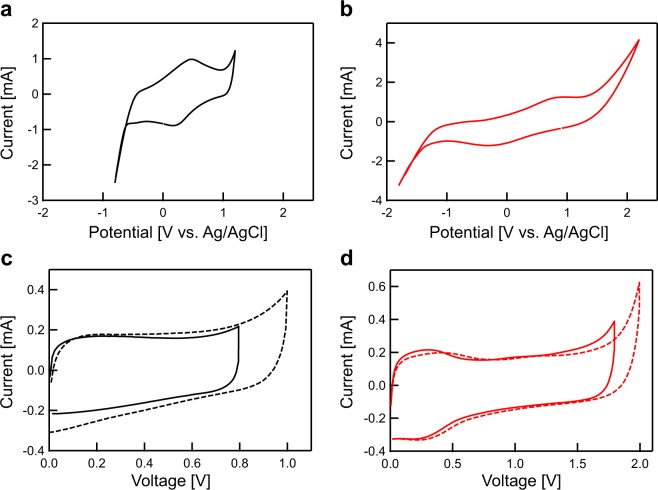
CVs in 1 M H_2_SO_4_ with (**a,b**) a three-electrode and (**c,d**) a symmetric two-electrode system: (**a,c**) AC and (**b,d**) BDND electrodes. The scan rate was 10 mV s^−1^.

The electrochemical properties of the BDND electrode were examined in 1 M H_2_SO_4_. According to the result of the CV measurement using a three-electrode system, the BDND electrode exhibited a wide potential window of about 3 V (Fig. 5b). Wide potential windows in aqueous electrolytes are a typical characteristic of BDD electrodes^6,33^. Therefore, the wide potential window obtained in this work for the BDND probably stems from the BDD part in the BDND. A redox peak pair around +0.3 V vs. Ag/AgCl can be attributed to the quinone/hydroquinone group existing in the edge site of the graphitic components^34,35^. Figure 5a illustrates the CV of an AC electrode, which exhibits a narrower potential window of 1.5 V than that of the BDND electrode. Similarly, a redox peak pair for the quinone/hydroquinone group was observed. Figure 5c,d display the CV obtained using a symmetric two-electrode system. The BDND electrode was found to exhibit a wide potential window of 1.8 V (Fig. 5d), whereas that of the AC electrode was 0.8 V in 1 M H_2_SO_4_ (Fig. 5c). The characteristics of the BDD are most likely responsible for the large cell voltage of the BDND in aqueous electrolyte. Although the BDND contained a large amount of sp^2^ carbon components, the potential window was found to be as wide as that of BDD thin film electrodes^6^. The double-layer capacitance of the BDND electrode (10 mV s^−1^) was calculated to be 15.1 F g^−1^, whereas that of the AC electrode was 20.4 F g^−1^. Figure S3 shows the CV of the as-deposited BDND and BDND after heat treatment for 8 h with a symmetric two-electrode system in 1 M H_2_SO_4_. The double-layer current was found to be 3.27 times larger for the heat-treated BDND than for the as-deposited BDND, which can be explained by the difference in the specific surface area of both BDNDs (Fig. 4a). Electrochemical impedance spectroscopy measurement was performed to estimate the electrochemical properties of the BDND electrode (Fig. S4). The Nyquist plot indicated a blocking behavior with a slope in the low frequency region, which is typical of conventional EDLCs.

**Figure 6:**
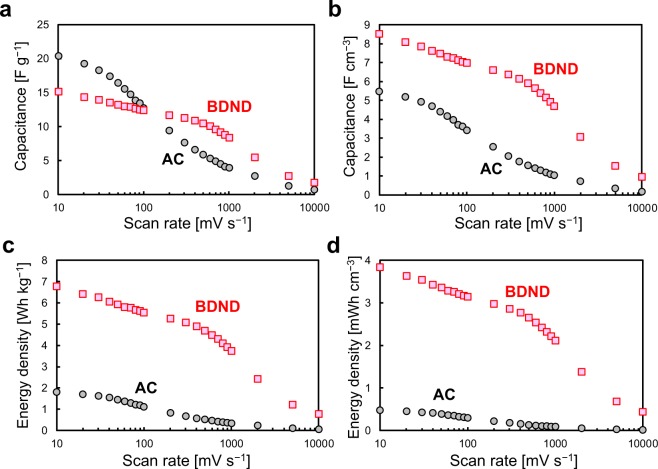
Capacitance (**a**) per unit weight of active material and (**b**) per unit volume of electrode layer; and energy density (**c**) per unit weight of active material and (**d**) per unit volume of electrode layer as a function of the scan rate. The capacitance was estimated from the CV data recorded in 1 M H_2_SO_4_ with a symmetric two-electrode system. The energy density was calculated according to the capacitance (panels a and b) and cell voltage (0.8 V and 1.8 V for AC and BDND, respectively).

The charge–discharge rate performance was investigated using CV with various scan rates (Fig. S5). Even at fast scan rates up to 1000 mV/s, the CVs for the BDND showed a constant current region. Figure 6a,b show the gravimetric and volumetric capacitance of the BDND and AC electrodes calculated from the CV data as a function of the scan rate using the formula *C* = *I*/*v*, where *C* is the capacitance, *I* is the charging current in the constant region, and *v* is the scan rate. The gravimetric capacitance of the BDND electrode was larger than that of the AC electrode when the scan rate was 100 mV/s or faster (Fig. 6a). From this result, the BDND electrodes can be considered to be suitable for high-rate charging–discharging. Since AC had well-developed micropores in the particles, its capacitance deteriorated for fast scan rates^36^. In contrast, the absence of such pores in the BDND allowed for the entire surface to contribute efficiently to the fast charging–discharging. The higher suitability of the BDND is further evinced when considering the volumetric capacitance (Fig. 6b) because the bulk density of the BDND electrode layer (0.52 g/cm^3^) was larger than that of the AC electrode layer (0.23 g/cm^3^)^27^. The energy density was calculated using the capacitance extracted from Figs. 6a,b according to the following equation: *E* = *CV*^2^/2/3600, where *E* is the energy density (in Wh/kg), *C* is the capacitance (in F/kg), and *V* is the cell voltage (in V). The cell voltage used for the calculation was 1.8 and 0.8 V for the BDND and AC, respectively. This difference caused the energy density of the BDND to be much larger than that of the AC (Fig. 6c,d). The large energy density of the BDND remained stable even at fast scan rates due to its relatively large capacitance of the BDND in the fast scan rate region (Fig. 6a,b).

**Figure 7:**
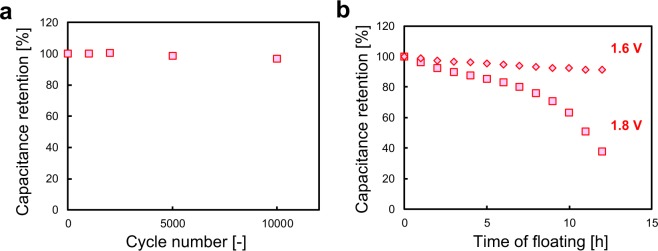
(**a**) Capacitance retention of the BDND electrode cell using 1 M H_2_SO_4_ as a function of the CV cycle number of the voltage cycling test. The cell voltage was scanned from 0 to 1.8 V at 10 mV/s. (**b**) Capacitance retention of the BDND electrode cell using 1 M H_2_SO_4_ as a function of time of floating. The cell voltage was held at 1.6 and 1.8 V during the test. The capacitance was estimated by CV at 10 mV/s.

To estimate the stability of the BDND electrode in repetitive charging–discharging cycles, a voltage cycle test was performed from 0 to 1.8 V at 10 mV/s. Figure 7a shows capacitance retention as a function of voltage cycle number, indicating sufficient stability of the electrode with a 3.5% loss of the capacitance after 10,000 cycles. The stability of the BDND electrode was also investigated with a floating test (Fig. 7b). When a cell voltage of 1.8 V was applied to the cell, the capacitance was found to decrease to 38% of the initial value after a floating time of 12 h. In the case of a cell voltage of 1.6 V, however, the capacitance was maintained at 91% of its original value. This result indicates that aqueous EDLCs having a BDND electrode and 1 M H_2_SO_4_ can be operated practically at a large cell voltage such as 1.6 V.

### Electrochemical investigation in saturated NaClO_4_

**Figure 8:**
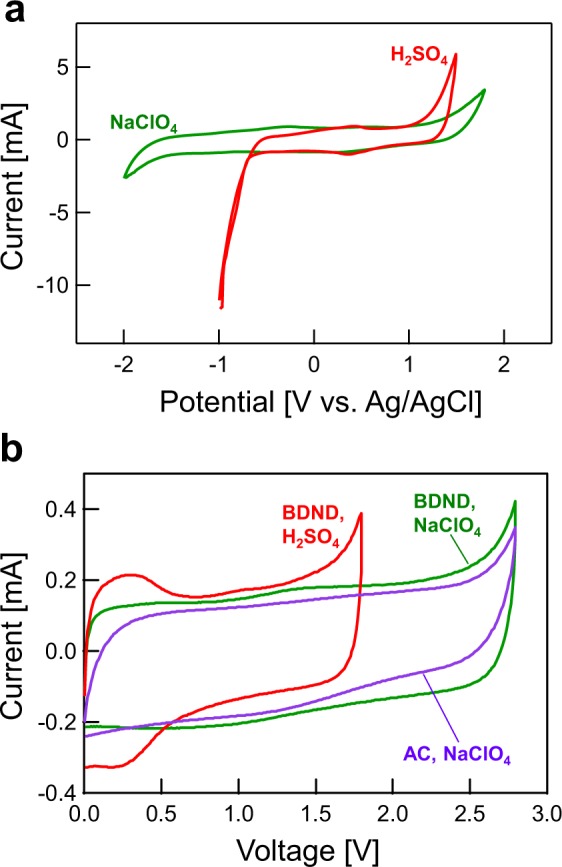
(**a**) CV in 1 M H_2_SO_4_ and saturated NaClO_4_ at the BDND electrode. The potential sweep rate was 10 mV s^−1^. (**b**) CV in 1 M H_2_SO_4_ and saturated NaClO_4_ with a symmetric AC and BDND two-electrode system. The scan rate was 10 mV s^−1^.

**Figure 9:**
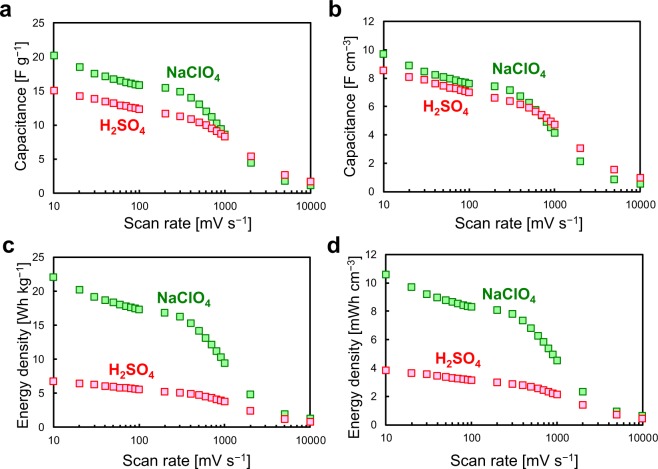
Capacitance (**a**) per unit weight of active material and (**b**) per unit volume of electrode layer; and energy density (**c**) per unit weight of active material and (**d**) per unit volume of electrode layer of the BDND electrode cell as a function of the scan rate.

Since the use of saturated NaClO_4_ as an aqueous electrolyte has been reported to expand the potential window^37^, we examined the electrochemical properties of the BDND electrode in saturated NaClO_4_ using CV with a three-electrode system (Fig. 8a). The potential window was confirmed to be wider for saturated NaClO_4_ than for 1 M H_2_SO_4_ at the BDND electrode. Consequently, the cell voltage in a two-electrode system was found to expand to 2.8 V in saturated NaClO_4_ at both BDND and AC electrodes (Fig. 8b). Figure 9 shows the capacitance of the BDND electrode in 1 M H_2_SO_4_ and saturated NaClO_4_ estimated from the CV recorded in a two-electrode system as a function of the scan rate. According to the result, the capacitance of the BDND electrode in saturated NaClO_4_ was similar to that in 1 M H_2_SO_4_. In contrast, the energy density of the BDND electrode cell with saturated NaClO_4_ was much higher than that exhibited in 1 M H_2_SO_4_ due to the larger cell voltage (Fig. 9c,d).

**Figure 10:**
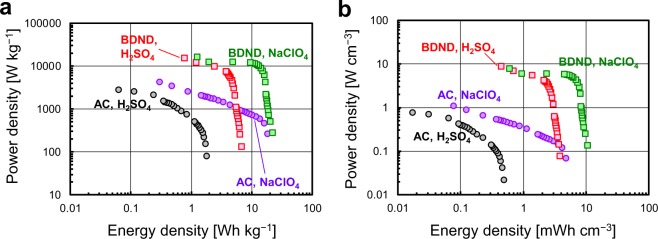
Ragone plots showing the power density vs. energy density (**a**) per unit weight of active material and (**b**) per unit volume of electrode layer of the AC and BDND electrode cell. The electrolytes used were 1 M H_2_SO_4_ and saturated NaClO_4_. The plot was created from a series of data at various scan rates shown in Figs. 6 and 9.

Figure 10 illustrates the Ragone plots showing the gravimetric and volumetric power density versus the energy density of the BDND and AC electrode cells using 1 M H_2_SO_4_ or saturated NaClO_4_ as electrolyte. For the cells using 1 M H_2_SO_4_, the BDND demonstrated significantly larger energy and power densities based on the large cell voltage. The energy density of the BDND electrode cell was further enhanced by using saturated NaClO_4_ as an aqueous electrolyte (approximately 20 Wh/kg with a power density of 10^2^–10^4^ W/kg or 0.01 Wh/cm^3^ with a power density of 0.1–10 W/cm^3^). Although the use of saturated NaClO_4_ improved the energy density of the AC electrode cell, the power density in the fast scan rate region was much lower than that of the BDND electrode cell. Therefore, the proposed aqueous supercapacitor using BDND as an electrode material is expected to be an energy storage device suitable for high-speed charging–discharging.

## Conclusions

Herein, a BDND was fabricated by depositing a BDD layer on a detonation ND as a substrate material using the MPCVD method toward application as electrode materials for aqueous EDLCs. Characterization with TEM and UV Raman spectroscopy indicated that a large amount of sp^2^ carbon components was deposited simultaneously during the CVD process. The sp^2^ carbon components could be removed partly by heat treatment resulting in a large specific surface area of 658 m^2^/g, which was about half of that of AC, i.e., 1318 m^2^/g. The electrochemical investigation of the BDND demonstrated its large capacitance of 15.1 F/g in 1 M H_2_SO_4_, which was close to that of AC, i.e., 20.4 F/g. In a symmetric two-electrode system with 1 M H_2_SO_4_, the BDND electrode exhibited a large working cell voltage of 1.8 V, which was much larger than that of the AC electrode, i.e., 0.8 V. The large cell voltage of the BDND electrode resulted in a significantly larger energy density than that of the AC electrode. Moreover, the decrease in the energy density at fast scan rates was remarkably suppressed in the BDND electrode in comparison to that of the AC electrode, indicating that the BDND electrode is suitable for fast charging–discharging. The use of saturated NaClO_4_ expanded the cell voltage to 2.8 V. These results led us to conclude that BDND electrodes are useful for the preparation of aqueous EDLCs that can exhibit a high energy density even in fast charging–discharging operations.

## Methods

The BDND was fabricated by depositing a BDD layer on a detonation ND (Daicel corporation, average diameter: 4.7 nm, specific surface area: 270 m^2^/g) via MPCVD with a microwave power of 1300 W, stage temperature of 800 °C, and deposition time of 8 h. The detailed MPCVD condition is described elsewhere^22,38^. A mixture of 70% trimethoxyborane/methanol and acetone (the B/C atomic concentration ratio was 20,000 ppm) was used as a carbon/boron source. After the MPCVD process, the sample was heated in air at 425 °C for 8 h to minimize the amount of sp^2^ carbon impurities, affording the BDND^39^.

Transmission electron microscopy (TEM, JEM-2100F, JEOL) and Raman spectroscopy (inVia Reflex, Ranishaw, *λ*_ex_ = 325 nm) were employed for the characterization of the BDND. The specific surface area was calculated according to the Brunauer–Emmett–Teller (BET) theory from the nitrogen gas sorption isotherms (BELSORP-max, MicrotracBEL). The electrical conductivity was evaluated from the linear slope of the *I*–*V* curve measured between both ends of the BDND packed into a glass tube with an inner diameter of 1.0 mm. Both ends of the packed BDND were pressed using copper rods with a diameter of 0.8 mm^40^. The BDND electrode was prepared by casting an ink containing BDND on a glassy carbon (GC) disk electrode (diameter: 3 mm)^27,41^. First of all, 10 mg of the BDND was dispersed in 0.5 mL of 30 wt% ethanol to prepare a BDND ink. Then, 20 μL of the BDND ink was cast on the GC electrode and dried at 60 °C. Finally, 10 μL of 5 wt% Nafion was coated to fix the BDND layer. In this way, the amount of active material coated on the GC electrode was calculated to be 0.4 mg and was found to be 0.37 ± 0.04 mg. Electrochemical investigation was conducted in 1 M H_2_SO_4_ and saturated NaClO_4_ aqueous solutions. The measurement in a three-electrode system was performed with an Ag/AgCl electrode and a platinum spiral wire as the reference and counter electrodes, respectively, which were connected to a potentio/galvanostat system (HZ-7000, Hokuto Denko). Additionally, the electrochemical measurement in a symmetric two-electrode system was performed using a beaker cell. An AC electrode was also prepared according to a procedure similar to that applied to the BDND electrode and using an ink containing 8.9 mg of AC (FUJIFILM Wako) and 1.1 mg of acetylene black as a conductive agent in 0.5 mL of 30 wt% ethanol.

## Supplementary information

Supplementary Information
