# Author Correction: Boron-doped Nanodiamond as an Electrode Material for Aqueous Electric Double-layer Capacitors

**DOI:** 10.1038/s41598-021-87509-z

**Published:** 2021-04-19

**Authors:** Kenjo Miyashita, Takeshi Kondo, Seiya Sugai, Takahiro Tei, Masahiro Nishikawa, Toshifumi Tojo, Makoto Yuasa

**Affiliations:** 1 Department of Pure and Applied Chemistry, Faculty of Science and Technology, Tokyo University of Science, 2641 Noda, Chiba, 278-8510 Japan; 2 Daicel Corporation, 1239 Shinzaike, Aboshi-ku, Himeji, Hyogo 671-1283 Japan

Correction to: *Scientific Reports* https://doi.org/10.1038/s41598-019-54197-9, published online 28 November 2019

The original version of this Article contained a calculation error in electric conductivity values. As illustrated in the Methods section, the inner diameter of the glass tube for the measurement was 1.0 mm. However, the authors incorrectly used 10.0 mm for the calculations. In addition, an incorrect equation $\upsigma =\frac{1}{R} \cdot \frac{A}{l}$ was used for calculation of conductivity. The correct equation $\upsigma =\frac{1}{R} \cdot \frac{l}{A}$ has now been used, where, *σ* is conductivity, *R* is electrical resistance measured, *A* is cross-sectional area of the cylinder, and *l* is length of the cylinder. As a result, the electric conductivity values in the original version of the Article were about 100 times smaller than the correct values.

Consequently, in the Abstract,

“The BDND comprises BDD and sp^2^ carbon components, and exhibits a conductivity above 10^−2^ S cm^−1^ and a specific surface area of 650 m^2^ g^−1^.”

now reads:

“The BDND comprises BDD and sp^2^ carbon components, and exhibits a conductivity above 1 S cm^−1^ and a specific surface area of 650 m^2^ g^−1^.”

In addition, in the Results and Discussion section, under the subheading ‘Preparation of the boron-doped nanodiamond’,

“The electrical conductivity of the as-deposited BDND was increased rapidly from 1.8 × 10^−9^ S cm^−1^ to 3.5 × 10^−4^ S cm^−1^ with a deposition time of 1 h. The BDND prepared with 8 h deposition time exhibited a conductivity sufficient for an electrochemical electrode material (2.0 × 10^−2^ S cm^−1^).”

now reads:

“The electrical conductivity of the as-deposited BDND was increased rapidly from 7.2 × 10^−7^ S cm^−1^ to 1.4 × 10^−2^ S cm^−1^ with a deposition time of 1 h. The BDND prepared with 8 h deposition time exhibited a conductivity sufficient for an electrochemical electrode material (4.0 S cm^−1^).”

Finally, the y-axis scale and conductivity value in Figure 1 was incorrect. The original Figure 1 and accompanying legend appear below.

**Figure 1:**
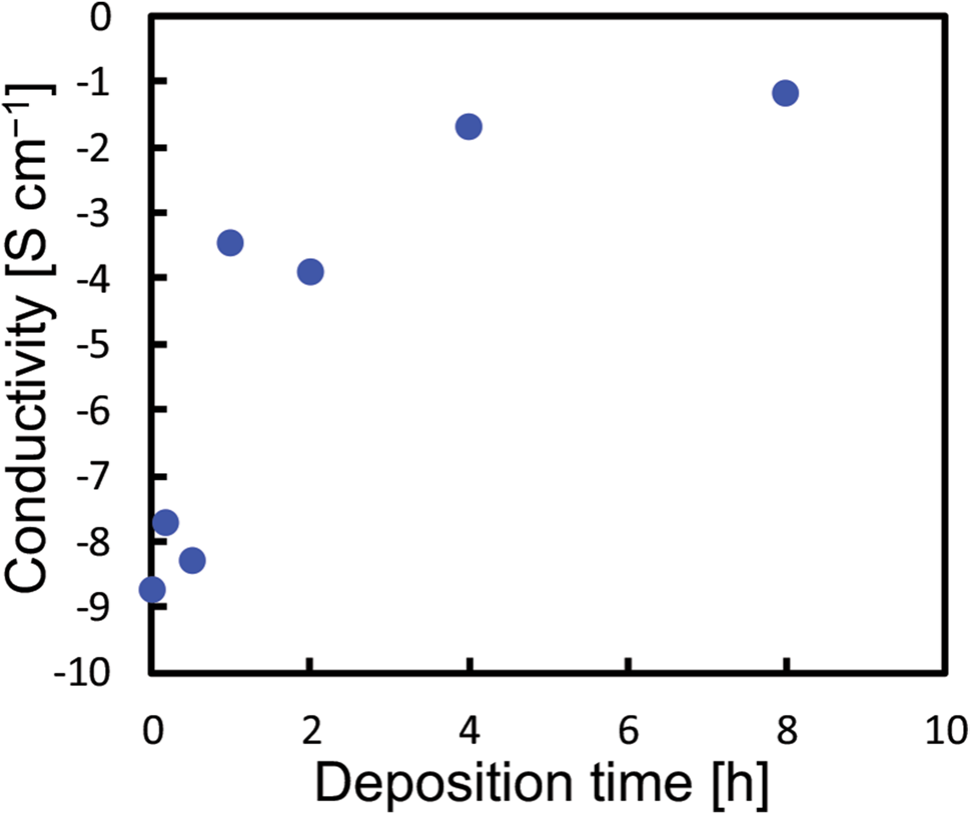
Electrical conductivity of the as-deposited BDND as a function of the CVD deposition time for the BDND preparation.

The original Article has been corrected.

