# Supplementary material for: Boron-doped Nanodiamond as an Electrode Material for Aqueous Electric Double-layer Capacitors
Source: Sci Rep. 2019 Nov 28;9:17846. doi: 10.1038/s41598-019-54197-9 (PMC6882838; doi:10.1038/s41598-019-54197-9)
Supplement: Supplementary file 1 — Supplementary Information [file 41598_2019_54197_MOESM1_ESM.pdf]

Supplementary Information for

Boron-doped Nanodiamond as an Electrode Material for Aqueous Electric Double-layer Capacitors

Kenjo Miyashita[1], Takeshi Kondo[1,*], Seiya Sugai[1], Takahiro Tei[2], Masahiro Nishikawa[2], Toshifumi Tojo[1], Makoto Yuasa[2]

[1]Department of Pure and Applied Chemistry, Faculty of Science and Technology, Tokyo University of Science, 2641 Noda, Chiba 278-8510, Japan

[2]Daicel Corporation, 1239 Shinzaike, Aboshi-ku, Himeji, Hyogo 671-1283, Japan

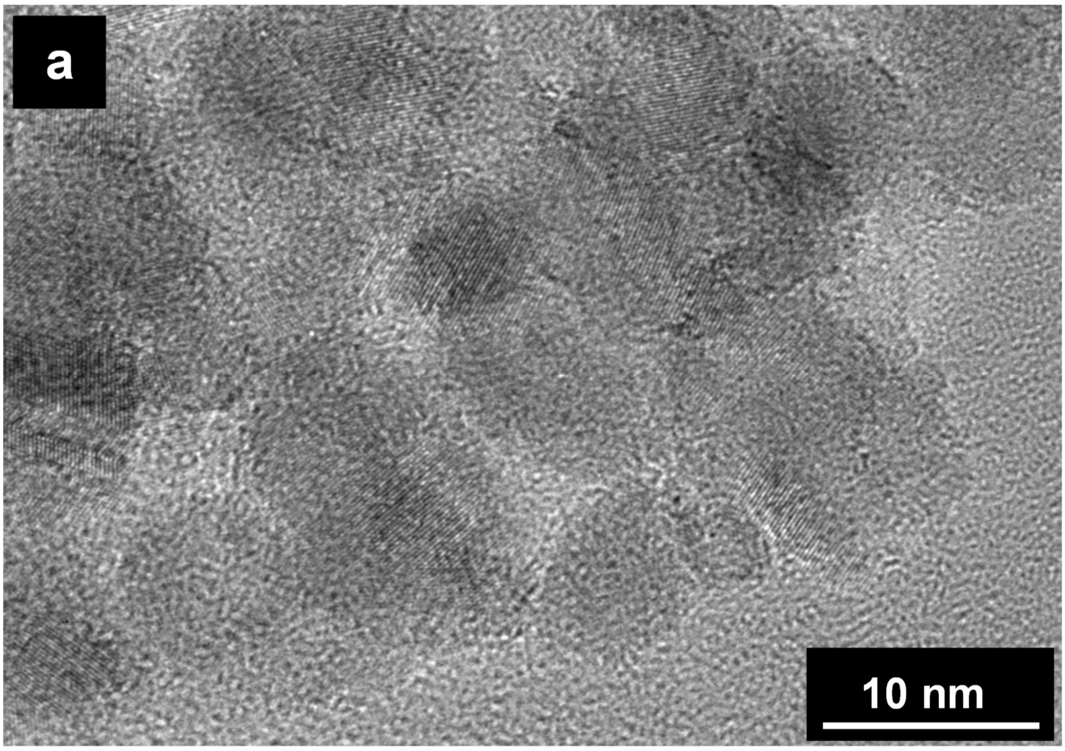

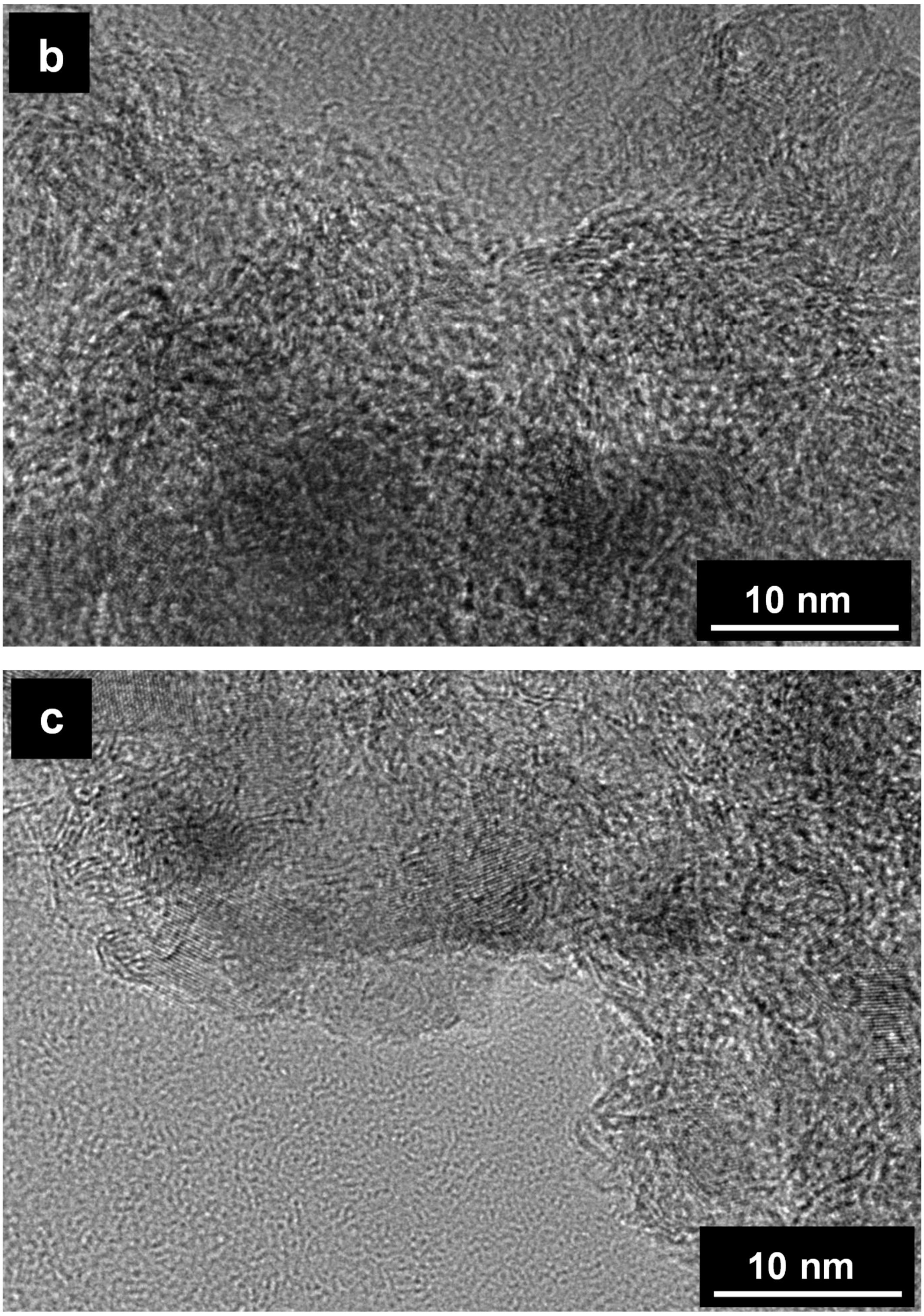

Figure S1. TEM images of the (a) ND, (b) as-deposited BDND, and (c) heat-treated BDND.

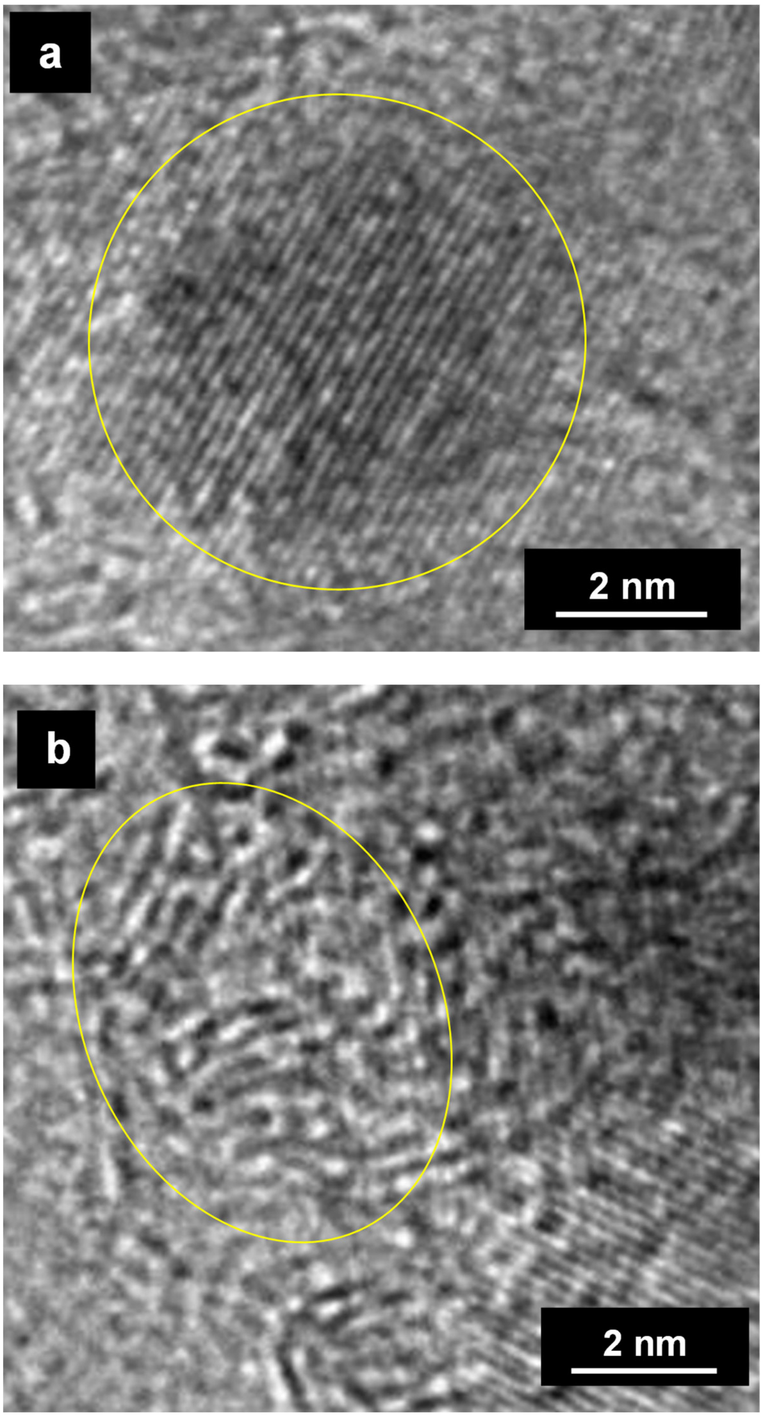

Figure S2. Magnified TEM images of (a) ND and (b) (heat-treated) BDND. Yellow circle indicates the presence of ND (a) and graphitic carbon (b) for estimation of interlayer spacing.

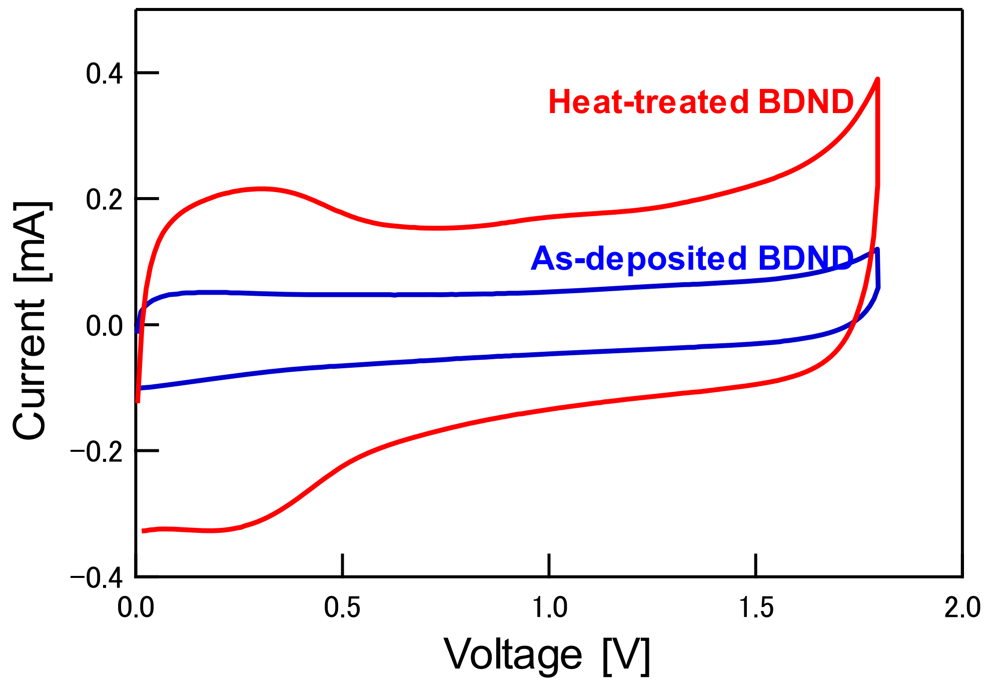

Figure S3. CVs in 1 M H_2SO_4 at as-deposited and heat-treated BDND electrodes with a symmetric two-electrode system. The scan rate was 10 mV s^{-1}.

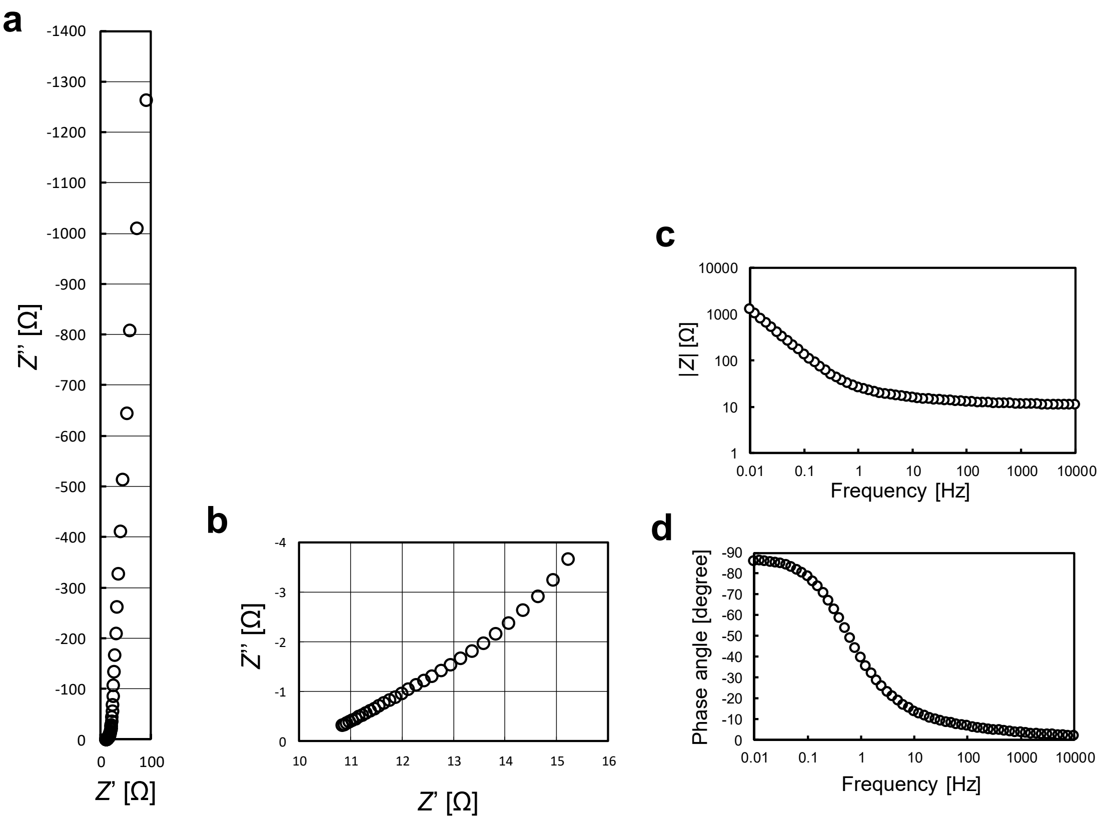

Figure S4. (a and b) Nyquist and (c and d) Bode plot for BDND electrode cell with 1 M H_2SO_4. Panel b indicates magnification of low frequency region of the Nyquist plot (panel a).

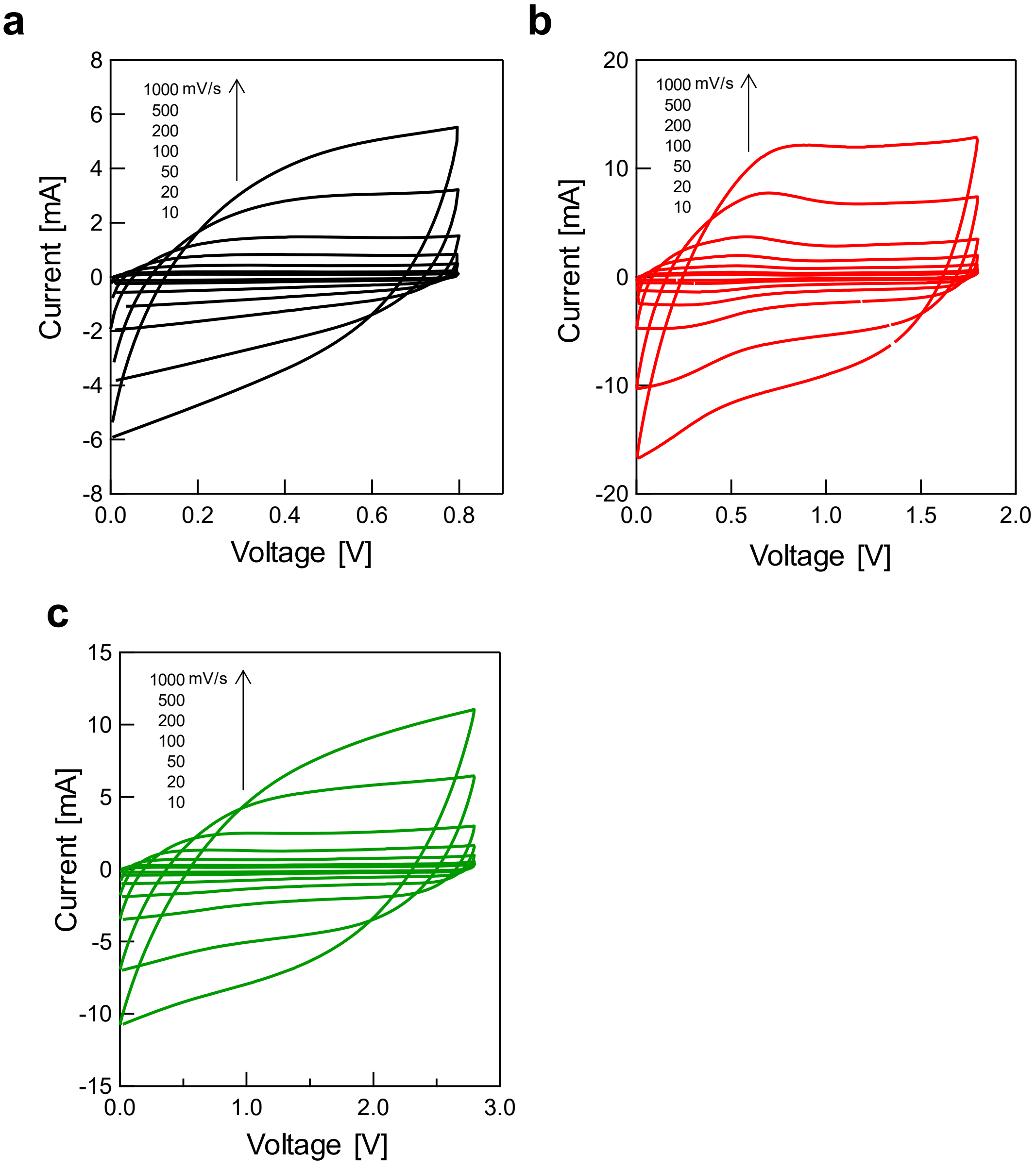

Figure S5. CVs in (a, b)1 M H_2SO_4 and (c) saturated $NaClO_4$ at (a) AC and (b, c) BDND electrodes with a symmetric two-electrode system. The scan rate was varied from 10 to 1000 mV s^{-1}.
